# Porto-spleno-mesenteric thrombosis secondary to medroxyprogesterone acetate as a treatment for abnormal uterine bleeding: a case report

**DOI:** 10.1093/jscr/rjaf791

**Published:** 2025-10-06

**Authors:** David J Alvarez Chavez, Javier A Maciel Urzua, Maria C Torres González, Carlos A Bautista López, Carla M Cruz Rocha, Esther A Casado De La Torre, Daniela I Sánchez Lozano

**Affiliations:** Department of General Surgery, Universidad de Guadalajara, Hospital Civil de Guadalajara “Dr. Juan I. Menchaca,” Calle Salvador Quevedo y Zubieta, 750, 44360 Guadalajara, Jalisco, México; Department of General Surgery, Universidad de Guadalajara, Hospital Civil de Guadalajara “Dr. Juan I. Menchaca,” Calle Salvador Quevedo y Zubieta, 750, 44360 Guadalajara, Jalisco, México; Department of General Surgery, Universidad de Guadalajara, Hospital Civil de Guadalajara “Dr. Juan I. Menchaca,” Calle Salvador Quevedo y Zubieta, 750, 44360 Guadalajara, Jalisco, México; Department of General Surgery, Universidad de Guadalajara, Hospital Civil de Guadalajara “Dr. Juan I. Menchaca,” Calle Salvador Quevedo y Zubieta, 750, 44360 Guadalajara, Jalisco, México; Department of General Surgery, Universidad de Guadalajara, Hospital Civil de Guadalajara “Dr. Juan I. Menchaca,” Calle Salvador Quevedo y Zubieta, 750, 44360 Guadalajara, Jalisco, México; Department of General Surgery, Universidad de Guadalajara, Hospital Civil de Guadalajara “Dr. Juan I. Menchaca,” Calle Salvador Quevedo y Zubieta, 750, 44360 Guadalajara, Jalisco, México; Department of General Surgery, Universidad de Guadalajara, Hospital Civil de Guadalajara “Dr. Juan I. Menchaca,” Calle Salvador Quevedo y Zubieta, 750, 44360 Guadalajara, Jalisco, México

**Keywords:** splanchnic thrombosis, mesenteric thrombosis, medroxyprogesterone, venous thromboembolism, contraceptive, case report

## Abstract

Acute mesenteric venous thrombosis is a rare and potentially lethal cause of intestinal ischemia. We report a 38-year-old woman with abnormal uterine bleeding, with anemia, and prolonged use of medroxyprogesterone acetate. She presented severe abdominal pain, peritoneal signs, and systemic inflammatory response. Computed tomography revealed thrombosis of the portal, splenic, and superior mesenteric veins with jejunal wall thickening. Initial laparotomy showed a viable bowel; full anticoagulation was started. She later developed perforation requiring jejunal resection and subsequent reoperation for anastomotic dehiscence. Extensive workup revealed no prothrombotic disorder other than progestin use. This case highlights the need to consider hormonal agents as potential triggers of porto-spleno-mesenteric thrombosis, even in young women without comorbidities, and to pursue multidisciplinary, individualized management to balance timely surgery with preservation of bowel length.

## Introduction

Acute mesenteric ischemia (AMI) is an uncommon but life-threatening surgical emergency with mortality rates of 60%–90% [1, 2]. While most cases are arterial, 5%–15% are due to venous thrombosis, and porto-spleno-mesenteric involvement is particularly rare [2]. Risk factors include cirrhosis, intra-abdominal malignancy, and hereditary or acquired prothrombotic states [2–4]. Hormonal contraceptives are recognized contributors, but reports linking medroxyprogesterone acetate (MPA) specifically to extensive splanchnic venous thrombosis are scarce. We present such a case in a previously healthy woman, emphasizing diagnostic challenges, surgical decision-making, and the relevance of identifying reversible etiologies.

## Case report

A 38-year-old woman with a history of abnormal uterine bleeding and anemia was treated with MPA and intermittent transfusions. She presented with 72 h of severe, diffuse abdominal pain (9/10), nausea, vomiting, and peritoneal signs. Laboratory evaluation revealed leukocytosis and elevated inflammatory markers.

**Figure 1:**
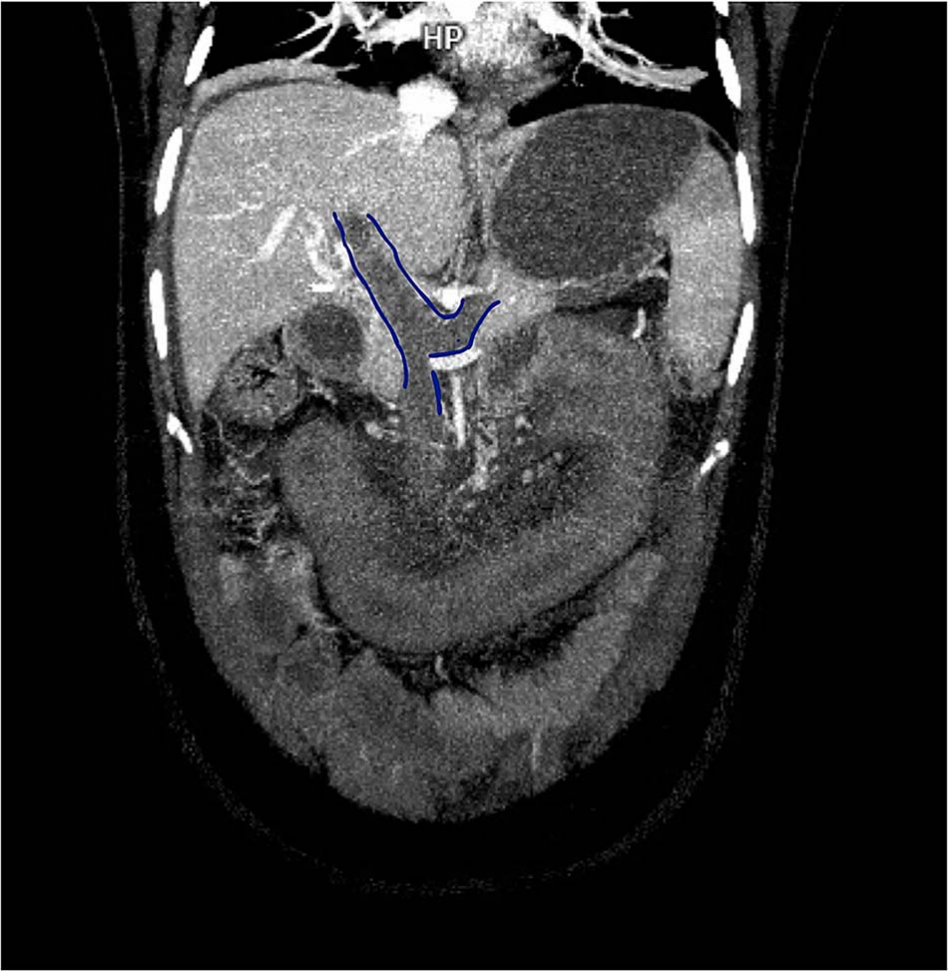
Contrast-enhanced CT in venous phase showing thrombosis of the portal, splenic, and superior mesenteric veins with proximal dilatation and thickening of the jejunal wall.

**Figure 2:**
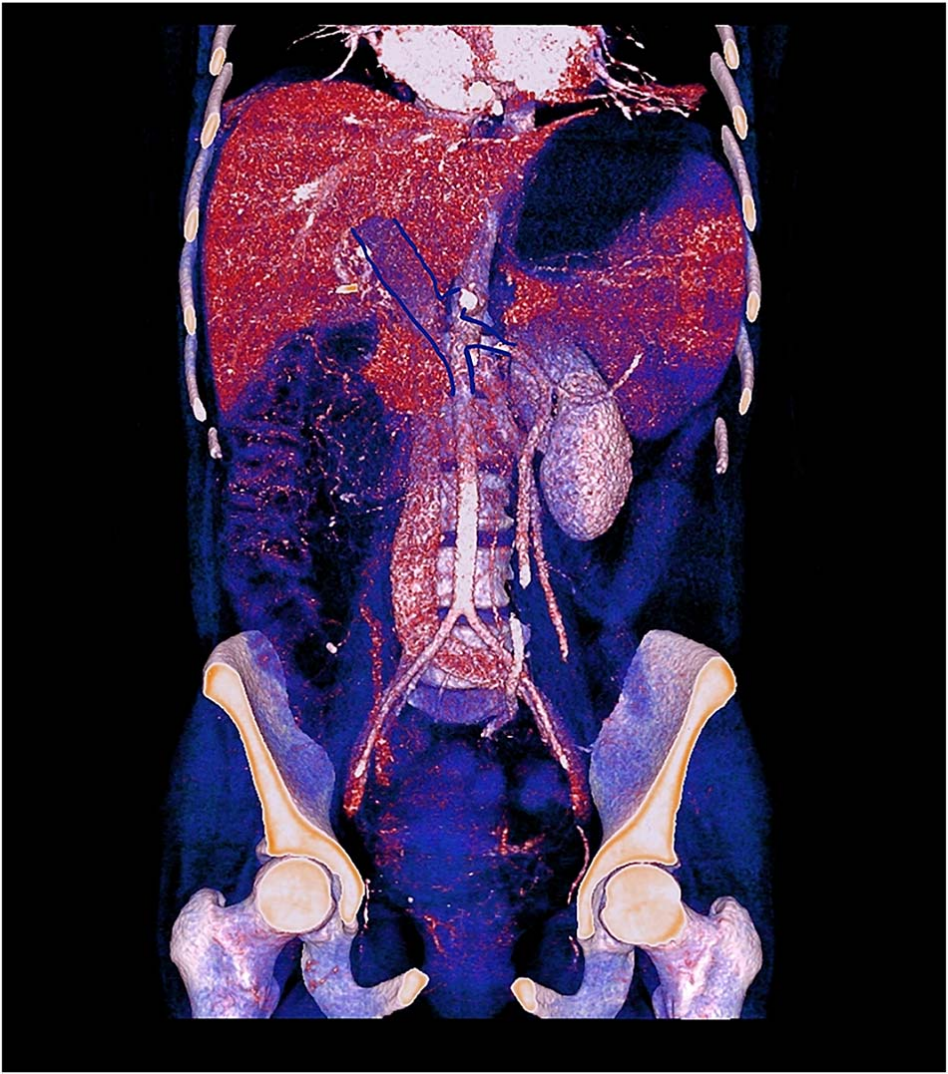
3D contrast-enhanced CT rendering reconstruction in venous phase showing thrombosis of the portal, splenic, and superior mesenteric veins with proximal dilatation and thickening of the jejunal wall.

Contrast-enhanced computed tomography (CT) demonstrated thrombosis of the portal vein, splenic vein, and superior mesenteric vein with proximal dilatation, diffuse jejunal thickening, mesenteric fat stranding, and mild splenomegaly (Figs 1 and 2). No pneumatosis or portal venous gas was seen.

Emergency laparotomy revealed a 60 cm jejunal segment with hyperemia and mesenteric edema, 15 cm distal to the ligament of Treitz, without necrosis. Angiology recommended anticoagulation without resection. Low-molecular-weight heparin was initiated; the patient was managed with bowel rest and supportive care.

On day 4, she presented with worsening pain, fever, and sepsis. Repeat CT showed persistent venous obstruction and mesenteric edema. Despite anticoagulation, 48 h later she developed pneumatosis and free air. In laparotomy re-exploration, jejunal perforation involving 90% of the lumen was found; 15 cm was resected with primary anastomosis.

Three days later, anastomotic leakage was identified. Revision surgery with re-anastomosis and nasojejunal feeding was performed. Histopathology revealed ischemic-hemorrhagic necrotic enteritis with non-necrotizing granulomatous reaction.

Extensive hematologic evaluation—including lupus anticoagulant, protein C/S, antithrombin III, and antiphospholipid antibodies—was negative, except for suspicion of antiphospholipid syndrome on initial testing which was later discarded. No other prothrombotic risk factors were identified apart from MPA use. She was discharged on rivaroxaban, nutritional support, and non-hormonal therapy for uterine bleeding.

## Discussion

Porto-spleno-mesenteric venous thrombosis represents a minority of AMI cases, accounting for ˂15%–20% of presentations, but its early recognition is crucial to prevent irreversible bowel injury [5, 6]. In our patient, the only identified risk factor was prolonged use of MPA, consistent with reports linking progestin-only contraception to an increased risk of venous thromboembolism, with a relative risk estimated at 2.67 [7–9]. Although most venous thromboses can be managed with anticoagulation alone, progression to transmural necrosis and perforation, as in our case, necessitates surgical intervention.

Previous reports highlight the heterogeneity of clinical evolution in mesenteric venous thrombosis. Aguilar Jaramillo *et al.* [2] described a young patient with mesenteric and portal venous thrombosis progressing to intestinal ischemia, emphasizing the diagnostic challenge in the absence of classic risk factors. Similarly, Andreu-Ruiz and Sanmartín-Monzó [3] reported idiopathic porto-mesenteric-splenic thrombosis treated with locoregional fibrinolysis, underscoring the potential role of endovascular approaches in carefully selected patients. By contrast, Haider *et al.* [8] documented a rare case of splenic artery thrombosis triggered by MPA use, which, although arterial, reinforces the association between progestin exposure and thrombotic events affecting the splanchnic circulation.

The management of venous mesenteric ischemia remains debated. Some authors argue that unnecessary laparotomy in patients with viable bowel may worsen outcomes, whereas delayed intervention risks perforation and sepsis [10]. Biomarkers such as D-dimer, lactate, and intestinal fatty acid–binding protein have been studied for early detection [11], but imaging—particularly contrast-enhanced CT—remains the gold standard for diagnosis [4, 5].

This case underscores the importance of identifying reversible and modifiable risk factors for thrombosis, especially hormonal therapy in young women, and highlights the need for multidisciplinary collaboration between surgery, radiology, hematology, and gynecology. While minimally invasive and endovascular strategies are increasingly reported [3–5], open surgery remains indispensable in unstable patients or when there is clinical suspicion of necrosis or perforation.
